# Noise Exposure Potentiates Exocytosis From Cochlear Inner Hair Cells

**DOI:** 10.3389/fnsyn.2021.740368

**Published:** 2021-09-29

**Authors:** Luis E. Boero, Shelby Payne, Maria Eugenia Gómez-Casati, Mark A. Rutherford, Juan D. Goutman

**Affiliations:** ^1^Instituto de Investigaciones en Ingeniería Genética y Biología Molecular “Dr. Héctor N. Torres” (INGEBI), Buenos Aires, Argentina; ^2^Instituto de Farmacología, Facultad de Medicina, Universidad de Buenos Aires, Buenos Aires, Argentina; ^3^Department of Otolaryngology, Washington University School of Medicine, St. Louis, MO, United States

**Keywords:** noise exposure, hair cells, exocytosis, *Vglut3* ^KO^, synapse loss

## Abstract

Noise-induced hearing loss has gained relevance as one of the most common forms of hearing impairment. The anatomical correlates of hearing loss, principally cell damage and/or death, are relatively well-understood histologically. However, much less is known about the physiological aspects of damaged, surviving cells. Here we addressed the functional consequences of noise exposure on the capacity of inner hair cells (IHCs) to release synaptic vesicles at synapses with spiral ganglion neurons (SGNs). Mice of either sex at postnatal day (P) 15–16 were exposed to 1–12 kHz noise at 120 dB sound pressure level (SPL), for 1 h. Exocytosis was measured by tracking changes in membrane capacitance (ΔCm) from IHCs of the apical cochlea. Upon IHC depolarization to different membrane potentials, ΔC_*m*_ showed the typical bell-shaped curve that mirrors the voltage dependence of Ca^2+^ influx, in both exposed and unexposed cells. Surprisingly, from IHCs at 1-day after exposure (d.a.e.), we found potentiation of exocytosis at the peak of the bell-shaped curve. The increase in exocytosis was not accompanied by changes in whole-cell Ca^2+^ influx, suggesting a modification in coupling between Ca^2+^ channels and synaptic vesicles. Consistent with this notion, noise exposure also changed the Ca^2+^-dependence of exocytosis from linear to supralinear. Noise exposure did not cause loss of IHCs, but did result in a small reduction in the number of IHC-SGN synapses at 1-d.a.e. which recovered by 14-d.a.e. In contrast, a strong reduction in auditory brainstem response wave-I amplitude (representing synchronous firing of SGNs) and distortion product otoacoustic emissions (reflecting outer hair cell function) indicated a profound hearing loss at 1- and 14-d.a.e. To determine the role of glutamate release in the noise-induced potentiation of exocytosis, we evaluated vesicular glutamate transporter-3 (*Vglut3*) knock-out (KO) mice. Unlike WT, IHCs from *Vglut3*^KO^ mice showed a noise-induced reduction in ΔC_*m*_ and Ca^2+^ influx with no change in the Ca^2+^-dependence of exocytosis. Together, these results indicate that traumatic noise exposure triggers changes of IHC synaptic function including a *Vglut3*-dependent potentiation of exocytosis.

## Introduction

Among the diverse etiologies of hearing loss, one of the most prevalent in modern societies is acoustic trauma, which is typically observed after the acute, or chronic, exposure to loud sounds (Śliwińska-Kowalska and Zaborowski, 2017). The anatomical consequences of noise exposure to the inner ear are varied and have been studied over decades, establishing several important consequences such as damage to mechanosensory hair cells and/or their stereocilia, auditory neurons and other specialized cells in the inner ear (Spoendlin, 1971; Liberman and Kiang, 1978; Robertson and Johnstone, 1980; Wang et al., 2002). The degree of damage, as well as the identity of the injured structures, depends on the intensity and the duration of the noise exposure.

Hair cell loss is particularly detrimental because they are the sensory cells in the inner ear, responsible for the detection and transduction of acoustic signals. Out of the two types of hair cells that co-exist in mammalian inner ears, outer hair cells (OHCs) have shown the highest sensitivity to noise exposure, especially at the basal turn (Spoendlin, 1971; Robertson and Johnstone, 1980; Wang et al., 2002). A critical characteristic of OHCs loss due to any cause (noise, ototoxic drugs, age) is the inability of mammalian sensory cells to regenerate (Edge and Chen, 2008), leading to a permanent elevation of hearing thresholds. While intense noise exposure leads to hair cell death, more moderate exposures that spare the cells still produce damage to structures such as the stereocilia of both OHCs and IHCs (Robertson and Johnstone, 1980; Liberman and Dodds, 1984; Saunders et al., 1985). For example, overstimulation of the hair bundle can disrupt or break the tip links, uncoupling the mechano-transduction channel complex from mechanical stimuli and leading to hair cell dysfunction (Pickles et al., 1987; Assad et al., 1991).

Synapses between inner hair cells (IHCs) and spiral ganglion neurons (SGNs), connecting the auditory periphery with the brain, are also sensitive to low or moderate sound overexposure. Massive swelling of SGN terminals at the contact point with IHCs has been shown to occur early after exposure, followed by a partial recovery within days (Spoendlin, 1971; Robertson, 1983). This phenomenon was prevented with the application of specific AMPA receptors antagonists during the noising protocol and could also be mimicked in experiments of acute perfusion of agonists in the absence of sound, suggesting a critical role for glutamate in producing damage to SGN terminals (Puel et al., 1991, 1998; Ruel et al., 2000, 2007). Moderate noise exposures that preserve hair cells and hearing thresholds may nonetheless reduce supra-threshold responses through immediate elimination of some IHC-SGN synapses followed by delayed SGN loss (Kujawa and Liberman, 2009). This noise induced synaptopathy was not observed in mice lacking the vesicular glutamate transporter type-3 (*Vglut3*^KO^), reinforcing the idea that synaptopathy is a form of glutamate excitotoxicity (Kim et al., 2019). Interestingly, in *Vglut3*^WT^ mice moderate noise exposure triggered synapse loss followed by partial recovery/regeneration as well as changes in synapse morphology, suggesting alterations in synapse function.

AMPA type receptors at SGN postsynaptic terminals generate excitation that is propagated to the central nervous system (CNS) (Matsubara et al., 1996; Ruel et al., 1999; Sebe et al., 2017). Multiple SGNs innervate each cochlear IHC forming individual synaptic contacts that are characterized by the presence of an intracellular extension of the presynaptic density called the synaptic *ribbon* to which synaptic vesicles are tethered (Matthews and Fuchs, 2010; Moser et al., 2020). Exocytosis of these glutamate-filled vesicles depends on Ca^2+^ influx through Ca_*V*_1.3 Ca^2+^ channels and is virtually inexhaustible due to efficient vesicle cycling (Moser and Beutner, 2000; Brandt et al., 2003; Johnson et al., 2005). A close “nanodomain” coupling between Ca_*V*_1.3 channels and synaptic vesicles has been demonstrated for synapses located in the apical region of the cochlea, whereas a more distant “microdomain” coupling has been described for mid-cochlear synapses (Brandt et al., 2005; Johnson et al., 2005, 2017). Functionally, this difference in physical coupling translates into a linear relation between exocytosis and Ca^2+^ influx for nanodomain coupling, and a supralinear relation for microdomain coupling.

To probe the immediate functional consequences of noise exposure to surviving and/or recovering ribbon synapses in IHCs, here we noise exposed juvenile mice just after hearing onset [postnatal day (P) 15–16] and then proceeded to count IHC-SGN synapses and compare synaptic exocytosis with patch-clamp recordings of membrane capacitance relative to unexposed littermates at one day after exposure (1 d.a.e.). Our noise exposure protocol (120 dB SPL, 1–12 kHz, for 1 h) targeted the apical end of the cochlea, where presynaptic exocytosis and postsynaptic transmission have been well-studied (Moser and Beutner, 2000; Glowatzki and Fuchs, 2002; Johnson et al., 2005; Goutman and Glowatzki, 2007), and where IHCs are relatively more accessible for patch-clamp recordings. Interestingly, our results show an enhancement of exocytosis in IHCs at 1 d.a.e. compared to unexposed cells, but unchanged Ca^2+^ currents. These findings were accompanied by a transient reduction in synapse number and a permanent elevation of hearing thresholds. Using *Vglut3*^KO^ mice, in which Ca^2+^-triggered release from IHCs is functional although glutamate is not concentrated in the synaptic vesicle lumen (Ruel et al., 2008; Seal et al., 2008), we also asked if glutamate release could play a role in the noise induced changes we observed. Our results add to the previously described histological modifications in the cochlea caused by noise exposure, indicating that IHCs can undergo acute functional changes that are important for understanding the phenomenon of acoustic trauma.

## Materials and Methods

### Animals

Mice were used in accordance with protocols approved by the Animal Studies Committee of Washington University in St. Louis as well as INGEBI and Facultad de Medicina, Universidad de Buenos Aires (UBA) Institutional Animal Care and Use Committee (IACUC) guidelines, and best practice procedures. *Vglut3* mice (Slc17a8^*tm*1E*dw*^) on C57BL/6J background were obtained from The Jackson Laboratory (RRID:IMSR_JAX:016931). Mice of either sex in a similar male/female ratio were used at postnatal age 15–22 (P15-P22).

### Acoustic Overexposure

Unrestrained mice were exposed to a 1–12 kHz band noise for 1 h at 120 dB sound pressure level (SPL) at P15. Mice were placed in individual cages on a suspended shelf in a custom-made acrylic chamber in which no sides were parallel. The sound stimulus was produced by an RX6 processor (Tucker-Davis Technologies, TDT), filtered (Frequency Devices, Inc.), amplified (Crown 75A power amplifier), and delivered to the acrylic 132 chamber *via* a speaker horn (JBL). The SPL was measured through a 1/4-inch free-field microphone (ACO Pacific) calibrated with a 124-dB SPL pistonphone (Bruel and Kjaer, Denmark). Prior to the experimental noise exposure, four quadrants of the chamber were sampled with the 1/4- inch microphone and sound pressure was confirmed to vary by no more than 0.5 dB SPL across these measurement positions.

### Cochlear Function Tests

Hearing function was assessed in unexposed animals at P16 (“Unexposed” group), 1-day after noise exposure (NE) and at 2-weeks after exposure. Auditory Brainstem Responses (ABR) were recorded from subcutaneous electrodes located at the vertex (active electrode) and behind the right pinna (reference electrode), with ground electrode placed on the back, under ketamine (100 mg/kg)/xylazine (20 mg/kg) anesthesia, using a sampling frequency of 25 kHz. ABR thresholds at frequencies presented in half-octave intervals (5.6, 8, 11.2, 16, 22.6, 32, 45.2 kHz) were determined using 5 ms tone pips (including 0.5 ms cosine^2^ rise/fall) at a repetition rate of 40 s^–1^. The responses were amplified (×10,000), filtered (100 Hz–3 kHz), and averaged using custom computer software (System 3; TDT). Stimuli were presented in 5 dB steps from 15 to 100 dB SPL in ascending order to the right ear. At each level, 1,024 responses were averaged, with stimulus polarity alternated. Response waveforms were rejected if the peak-to-peak voltage exceeded 15 μV. Threshold was defined as the lowest sound level at which a recognizable waveform was present. Waveforms were confirmed by their larger amplitudes and decreasing latencies at increasing stimulus levels. If hearing threshold was not detected at 100 dB SPL, the threshold value was assigned as >100 dB. For wave-I measurements, ABRs were evoked with tone pips presented at a rate of 21 s^–1^ and measured using TDT^TM^ hardware (Tucker Davis) in conjunction with BiosigRZ software (TDT). Responses to 500 stimulus presentations at each level were used to construct average ABR waveforms. ABR wave-I amplitudes were quantified offline at 8 and 22.65 kHz as the difference between the pre-stimulus baseline and the first positive peak.

Distortion Product Otoacoustic Emissions (DPOAEs) were recorded from the right ear using Emav software (S. Neely, Boys Town National Research Hospital) in conjunction with TDT^TM^ and custom hardware, with a sampling rate of 192 kHz. We measured cochlear emissions at 2f_1_-f_2_, using 8 and 22.65 kHz as f_2_ frequencies. The f_1_/f_2_ ratio was 1.22 and the f_2_ level 10 dB lower than the f_1_ level. The DPOAE threshold was defined as the lowest f2 level in which the signal-to-noise floor ratio is >1.

### Isolation of the Organ of Corti and Electrophysiological Recordings

Apical cochlear explants were isolated from P16-17 exposed and unexposed mice using the following dissection solution (in mM): NaCl 155, KCl 5.8, CaCl_2_ 1.3, MgCl_2_ 0.9, NaH_2_PO_4_ 0.7, D-glucose 5.6, HEPES 10, pH 7.4, 295–305 mOsm. This solution was also used for patch-clamp recordings. After removing the tectorial membrane, organ of Corti sections were placed in a chamber for electrophysiological recordings mounted on the stage of a Zeiss Axioskop FS microscope and viewed with differential interference contrast (DIC) using a 40X water-immersion objective and a camera with contrast enhancement Leica Mc120 HD (Leica, Germany). Preparations were used within 2 h. Tissue was continuously perfused with fresh extracellular solution. Recording pipettes were fabricated from 1 mm borosilicate glass and Sylgard coated. Electrode resistances in the recording solutions were typically 5.5–6.5 MΩ. Once whole-cell configuration was obtained, the preparation was perfused with an extracellular solution containing TEA 30 mM to inhibit potassium currents. Intracellular solution had the following composition (in mM): CsMeSO_3_ 115, TEA 13, MgCl_2_ 6, CaCl_2_ 0.4, EGTA-Cs 1, HEPES 5, Na_2_ATP 5, NaGTP 0.3, phosphocreatine 5, pH 7.2, 283–290 mOsm. Currents were recorded 5 min after whole-cell configuration was established using an EPC-10 patch-clamp amplifier driven by PatchMaster software (HEKA Electronics, Germany). Holding potentials were not corrected for liquid junction potentials. Recordings were made at room temperature (22–25°C).

### Membrane Capacitance Measurements and Analysis

Membrane capacitance was measured using a software-based method of a Lock-in amplifier (Patchmaster, HEKA), combined with compensation of pipette and resting cell capacitances by the EPC-10 circuitries. 1-kHz, 50-mV peak-to-peak sinusoid waves were applied for 400 ms at a DC holding potential of −80 mV before and after the test pulse. The sine wave was small enough to not activate any significant membrane current since accurate membrane capacitance calculation requires a high and constant membrane resistance (Rm). The capacitance signal from EPC10 was filtered at 5 kHz and sampled at 50 kHz. The mean Cm of IHCs was 10.63 ± 0.40 pF (random sample of 34 cells) and mean holding current at −70 mV was −49.49 ± 3.32 pA. Cells in which holding current exceeded −100 pA or access resistance was higher than 15 MΩ were excluded from the analysis.

IHC changes in membrane capacitance (ΔCm) and currents were analyzed off-line with custom-written routines in IgorPro 6.37 (Wavemetrics). ΔCm was estimated as the difference of the mean Cm after the test pulse (skipping the initial 40 msec) and the mean of prepulse Cm. Post-stimulus endocytosis was not observed. For leak subtraction of IHC currents, IHC membrane resistance was calculated from voltage steps between −80 and −60 mV. Ca^2+^ charge (QCa^2+^) was calculated as the integral of the Ca^2+^ current from its onset time to the end of the pulse (i.e., not including the tail current).

### Immunofluorescence, Confocal Microscopy, Synaptic Counts, Volume, and Intensity Measurements

P16-17 exposed and unexposed mice were sacrificed and temporal bones were collected for immunostaining. Cochleae were fixed in PFA 4% for 30 min and decalcified in EDTA 0.12 M for 15 min. Microdissected pieces were then blocked overnight in 5% normal donkey serum at room temperature. CtBP2 mouse (BD Biosciences; RRID:AB_399431), Ca_*V*_1.3 rabbit (Alomone Labs; RRID:AB_2039775), GluA3 goat (Santa Cruz Biotechnology; RRID:AB_2113895) and Myo7a rabbit (Proteus Biosciences; RRID:AB_2314838) primary antibodies were used with the appropriate Alexa Fluor-conjugated secondary antibodies (Life Tech.) as previously described (Jing et al., 2013; Sebe et al., 2017; Kim et al., 2019). Samples were batch processed using the same reagent solutions in six cohorts, each including exposed and unexposed WT and *Vglut3* KO mice.

Confocal stacks were collected without saturation of pixel intensity and sampled with a Z-step of 0.38 μm and pixel size of 50 nm in X and Y, on a Zeiss LSM 700 with a 63 × 1.4 NA oil objective lens. For quantitative analysis of IHC synapses, images were collected at the cochlear region delimited by the tonotopic characteristic frequencies of 8–12 KHz (Müller et al., 2005). Image stacks were imported to Imaris software (Bitplane) where the different labeled puncta in the IHC region were segmented as “surface” objects, using identical settings for each image stack including the “local contrast background subtraction” algorithm for calculating threshold. This built-in automatic thresholding algorithm compensates for differences in overall luminance between stacks and eliminates potential subjective bias of setting a user-defined arbitrary threshold value. Synapses were identified as juxtaposed pairs of presynaptic ribbons (labeled with anti-CtBP2) and postsynaptic AMPA-type glutamate receptor puncta (labeled with anti-GluA3). The total number of synapses, as well as the total number of CtBP2, GluA3, and Ca_*V*_1.3 puncta per z-stack were counted and then divided by the number of IHCs in the image. The volume of each labeled punctum was obtained directly from Imaris.

In the 14 days after exposure experiments, anti-Ca_*V*_1.3 was replaced with anti-Myosin7a in order to perform OHC counting. In this case, images were acquired at different cochlear locations spanning the whole cochlea. We counted the number of missing OHCs in each stack and consequently calculated the percentage of OHC survival per stack.

### Statistical Analysis

Data were tested for normality with the Shapiro-Wilk test or assumed to be not normally distributed due to sample size. Parametric or non-parametric tests were applied as appropriate. Data that passed normality tests is shown as mean ± standard error of the mean (SEM), whereas median ± interquartile range was employed for not normally distributed data. ABR and DPOAE data were compared using Kruskal-Wallis non-parametric tests followed by Dunn’s post-tests. OHC survival as well as the quantification of synaptic elements and volumes in control and exposed mice were compared using Mann-Whitney tests. Two-way ANOVA followed by Holm-Sidak multiple comparisons test were employed to compare changes in membrane capacitance, peak of calcium currents and the integral of the calcium currents. ANCOVA analysis was utilized to compare the linear fits of the Log ΔCm vs. Log QCa^2+^ data. N is the number of animals or cochleae, as indicated in each figure legend. Except for the Mann-Whitney test used to compare % OHC survival at each cochlear location, all statistical tests were 2-sided and evaluated at the α level of 0.05 in R Statistical Software (RRID:SCR_001905). Graphs were plotted in RStudio and in GraphPad Prism 8 (GraphPad, RRID:SCR_002798).

## Results

### Noise Exposure Produces Auditory Threshold Elevation, Outer Hair Cell Damage, and Inner Hair Cell Synapse Loss

**FIGURE 1:**
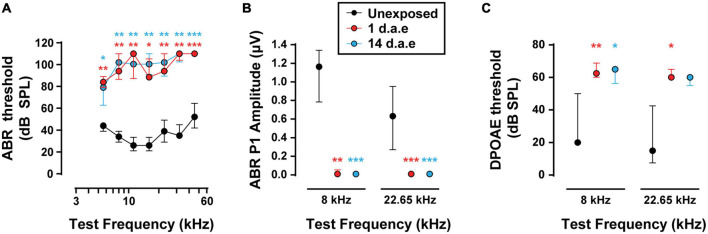
Exposure to 120 dB sound pressure level (SPL) noise at P15 induces a severe reduction in hearing sensitivity in P16-17 mice. **(A)** Auditory Brainstem Responses (ABR) thresholds for P16-17 control (Unexposed), P16-17 1 day after exposure (1 d.a.e.) and P29-30 14 days after exposure (14 d.a.e.) mice. **(B)** ABR wave-I baseline to peak amplitudes at 80 dB SPL for 8 and 22.65 kHz. **(C)** Distortion Product Otoacoustic Emission (DPOAE) thresholds in the same experimental groups for 8 and 22.65 kHz. Noise exposure triggers an elevation of cochlear thresholds which lasts for at least 14 days. In all cases, median ± interquartile ranges (IQRs) are shown, and the comparisons were made by Kruskal-Wallis non-parametric ANOVA followed by Dunn’s post-test (*p < 0.05, **p < 0.01, ***p < 0.001).

Hearing sensitivity was evaluated by ABR recordings before and after exposing awake mice at P15 to P16 to a broadband loud noise (Figure 1A, 1–12 kHz, 120 dB SPL for 1 h). With this protocol we aimed at producing acoustic trauma to the most apical area of the cochlea in animals at the beginning of their third postnatal week, provided that IHCs physiological properties are best characterized at this region and age range (Moser and Beutner, 2000; Brandt et al., 2003, 2005; Johnson et al., 2005; Ruel et al., 2008). As is shown in Figure 1A, we found a significant increase in ABR thresholds 1 day after exposure (1 d.a.e.) across all tested frequencies that lasted for at least 2 weeks after exposure (14 d.a.e.) being both groups compared to P16-17 unexposed mice (n_*Unexposed*_ = 5, n_1d__.a.e._ = 8, n_1__4d__.a.e._ = 8, see Supplementary Table 1 for the *p*-values of the different comparisons). Additionally, the analysis of suprathreshold ABR peak 1 amplitudes (Figure 1B), which reflect the summed sound-evoked activity of SGNs, showed a sharp reduction 1 d.a.e. (*p* = 0.0013 at 8 kHz; *p* = 0.0004 at 22.65 kHz, 80 dB SPL). This decrease in the amplitude of the sound-evoked response also remained 14 days after noise exposure (14 d.a.e.) (*p* = 0.0004 at 8 and 22.65 kHz), suggesting permanent threshold shift.

**FIGURE 2:**
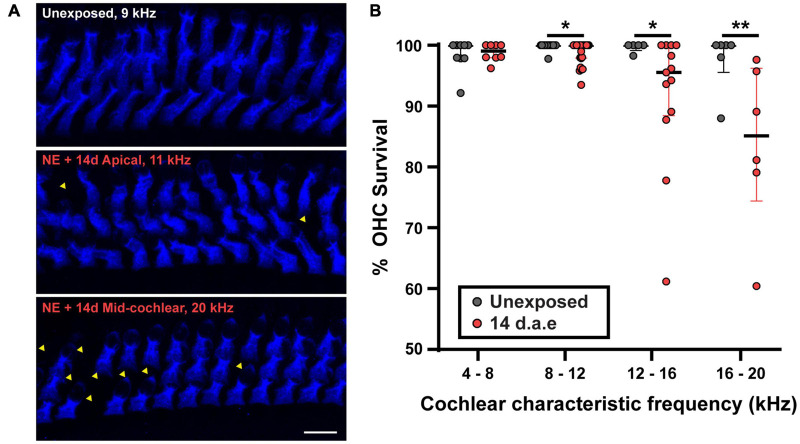
A small reduction in the number of outer hair cells (OHCs) was detected after exposure to loud noise for apical and mid-cochlear regions. **(A)** Representative images of the OHC region of cochlear turns from control (Unexposed) and 14 d.a.e. mice with MyoVIIa immunostaining. Top and middle panels correspond to images obtained from the cochlear region that has a characteristic frequency of 11 kHz for Unexposed and 14 d.a.e., respectively. Picture in lower panel was collected from the 20 kHz region from a 14 d.a.e. mice. Scale bar: 10 μm. **(B)** Percentage of OHC survival per z-stack for different cochlear segments. In the noise-exposed tissue there is a significant but small reduction in OHC survival for cochlear regions above 8 kHz. In all cases, median ± IQRs are shown, and the comparisons were made by Mann-Whitney unpaired test. *p < 0.05, **p < 0.01.

To address the impact of our noise-exposure protocol into the functional integrity of OHCs we measured DPOAE, the sound-evoked otoacoustic emissions generated by these cells. We detected an elevation of DPOAE thresholds in mice at 1 and 14 d.a.e. (Figure 1C, *p* = 0.0098 1 d.a.e. at 8 kHz; *p* = 0.0194 1 d.a.e. at 22.65 kHz; *p* = 0.0232, 14 d.a.e. at 8 kHz, same sample sizes than for ABRs). Therefore, the reduction in hearing sensitivity after noise exposure was at least partially due to dysfunction of OHC-driven cochlear amplification, leading to a reduced sound-driven excitation of IHC synapses at low sound levels. Provided this strong rise in DPOAE threshold, the integrity of OHCs in the 14 d.a.e. tissue was evaluated by myosin7a cochlear whole mount immunostaining, which specifically stain sensory cells of the inner ear (Figure 2). Representative pictures of the OHC region from P29 mice, either unexposed or 14 d.a.e., showed a slight reduction of OHC number in the apical region of noise-exposed cochleae, with a greater loss in the mid-basal region (Figure 2A). As can be seen in Figure 2B, noise-induced OHC degeneration followed a basal to apical gradient, showing a reduction of 14.15% in OHCs in regions corresponding to 16–20 kHz, 7.87% at 12–16 kHz, 1.03% at 8–12 KHz, but no significant change for 4–8 kHz (*p* = 0.0065 for the 16–20 kHz region, n_*Unexposed*_ = 6 images from 2 mice, n_1__4d__.a.e._ = 6 images from 3 mice, *p* = 0.0229 for the 12–16 kHz region, n_*Unexposed*_ = 5 images from 3 mice, n_1__4d__.a.e._ = 13 images from 7 mice, *p* = 0.0276 for the 8–12 kHz region, n_*Unexposed*_ = 14 images from 4 mice, n_1__4d__.a.e._ = 25 images from 8 mice, *p* > 0.05 for the 4–8 kHz region, n_*Unexposed*_ = 9 images from 4 mice, n_1__4d__.a.e._ = 8 images from 5 mice).

**FIGURE 3:**
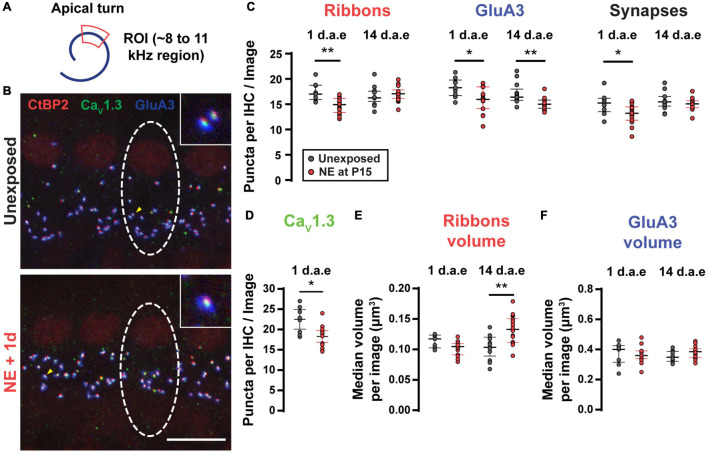
Noise exposure triggered a small but reversible synapse loss and a persistent increase in ribbon volume. **(A)** Scheme of the apical cochlear turn. Red area indicates the scanned region. **(B)** Representative images from Unexposed (upper panel) and 1 d.a.e. (lower panel) inner hair cells (IHCs). White dashed lines indicate the approximate contour of one IHC. Scale bar = 10 μm. **(C)** Mean number of Ribbons (CtBP2 positive puncta), GluA3 patches and Synapses (double positive puncta) per IHC in each confocal image for cochleae collected 1 and 14 days after exposure at P15 (1 and 14 d.a.e, respectively). **(D)** Mean number of Ca_*V*_1.3 positive puncta per IHC for unexposed and 1 d.a.e mice. Median volumes per stack for Ribbons **(E)** and GluA3 **(F)** for the four experimental groups. Line and whiskers indicate the median and the interquartile range, respectively. *p < 0.05, **p < 0.01, Mann-Whitney test.

It is well-established that moderate to loud noise exposures to adult rodents produces a sudden loss of synaptic contacts between IHC and SGNs, leading to reduced excitation of type I SGNs (Kujawa and Liberman, 2009, 2015; Kim et al., 2019; Hickman et al., 2020, 2021). To ask if our more intense, lower frequency noise protocol (Figure 3A) also produced alterations of IHC-SGN contacts, we performed synaptic counts in P16-17 control and noise-exposed animals (1 d.a.e.). Figure 3B shows representative images of a group of IHCs marked with antibodies against CtBP2-Ribeye, the major component at the presynaptic ribbon (Khimich et al., 2005; Rutherford, 2015); GluA3, a postsynaptic AMPA-type glutamate receptor subunit (Matsubara et al., 1996; Meyer et al., 2009; Liberman et al., 2011) and Ca_*V*_1.3 to label the voltage-gated calcium channels (Frank et al., 2010; Wong et al., 2014). Juxtaposed pairs of presynaptic ribbons (labeled with anti-CtBP2) and postsynaptic AMPA-type glutamate receptor puncta (labeled with anti-GluA3) were taken as a structural sign of a functional synapse, and therefore used as criteria for the identification of IHC-SGN synapses (Khimich et al., 2005; Liberman et al., 2011; Rutherford, 2015). Focusing the analysis to the 8–12 kHz region of the apical turn of the cochlea that was used for electrophysiological recording (see below), in control conditions an average number of 15.07 ± 0.72 synapses per IHC were observed (*n* = 9 images from 3 mice). One day after noise exposure we observed a reduction in the synapses count with an average of 13.05 ± 0.50 per IHC (Figure 3C, *p* = 0.0262, *n* = 15 images from 5 mice). Figure 3C also reveals that 1 day after exposure there was a decrease in both the number of ribbon puncta per IHC (average values of 17.37 ± 0.60 for unexposed mice and 14.72 ± 0.39 for 1 d.a.e mice, *p* = 0.0007) and GluA3 patches per IHC (average values of 18.25 ± 0.63 for unexposed mice and 15.8 ± 0.63 for 1 d.a.e mice, *p* = 0.0253). The number of Ca_*V*_1.3 labeled puncta were also computed per IHC in both control and exposed cochleae. We observed a significant reduction in the number of Ca_*V*_1.3 spots 1 d.a.e., from 22.51 ± 1.05 in controls to 18.29 ± 0.67 in exposed ears (*p* = 0.0101), representing a reduction of 19.75% in Ca_*V*_1.3 clusters (Figure 3D).

Two weeks after noise exposure (14 d.a.e., P29-30) the number of synapses and ribbons showed a complete recovery (synapse counts: 15.58 ± 0.47 in unexposed mice, 14.98 ± 0.26 in exposed mice, *p* = 0.26; ribbon counts: 16.63 ± 0.51 in unexposed mice, 17.05 ± 0.34 in exposed mice, *p* = 0.41, n_*Unexposed P*29–30_ = 13 images from 4 mice, n_14d.a.e_ = 18 images from 8 mice) (Figure 3C). On the contrary, the number of GluA3 patches did not come back to control values when evaluated 14 d.a.e. (unexposed mice at P29-30: 17.06 ± 0.57 in P29-30, 14 d.a.e. 15.10 ± 0.27, *p* = 0.0008) (Figure 3C). This mismatch in the number of recovered synapses and GluA3 puncta at 14 d.a.e. can be explained considering that: (i) as indicated before, the total number of GluA3 puncta is always larger than the number of synapses, i.e., some GluA3 puncta can be found not co-localizing to presynaptic elements (ribbonless synapses) (same for the reciprocal relation between ribbon and GluA3 puncta); (ii) at 14 d.a.e. there is a relatively larger loss of ribbonless synapses than those found co-localizing with ribbons (i.e., forming synapses) (1.57 ± 0.22 in unexposed P29-30 and 0.63 ± 0.09 in 14 d.a.e., *p* = 0.0001). It should also be noted that during development there is a pruning of postsynaptic terminals occurring over the first few postnatal weeks (Nemzou et al., 2006; Wong et al., 2014) that could be accelerated by noise exposure.

It has been shown that noise exposure can also affect the volume of ribbons and AMPA receptor patches in adult mice (Liberman and Liberman, 2015; Paquette et al., 2016; Kim et al., 2019), thus we sought to determine if these noise-induced alterations also took place with our exposure protocol. Therefore, the median volumes for ribbons (Figure 3E) and GluA3 (Figure 3F) were analyzed per stack for the four experimental groups mentioned above. We found no difference in the volumes of either ribbons or GluA3 patches after exposure except at 14 d.a.e. when ribbon volume was larger (average values of 0.10 ± 0.01 μm^3^ for unexposed mice and 0.13 ± 0.01 μm^3^ for exposed mice, *p* = 0.0014).

Taken together, results in Figures 1–3 confirmed that our noise exposure protocol produced a permanent increase in ABRs and DPOAES thresholds, and a transient reduction in the number of paired synapses between IHCs and SGNs.

### Potentiated Inner Hair Cell Exocytosis After Noise Exposure

**FIGURE 4:**
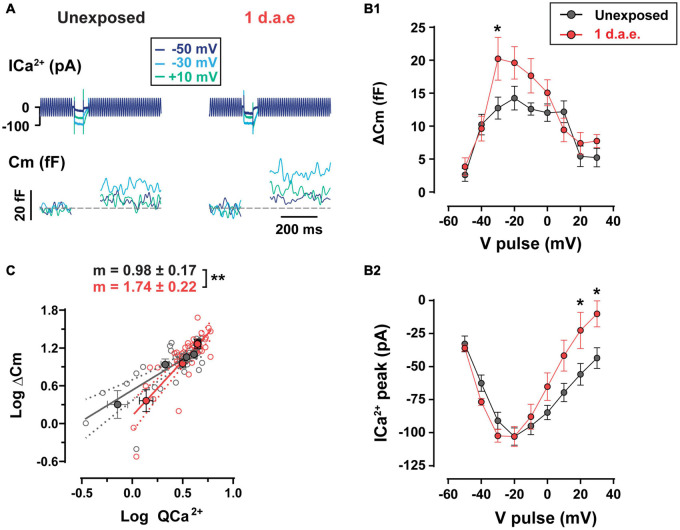
Increased exocytosis and altered Ca^2+^-dependence of release were found in noise-exposed inner hair cells (IHCs). **(A)** Calcium currents (ICa^2+^) and membrane capacitance (Cm) traces of representative control (Unexposed) and 1 d.a.e. IHCs elicited by 50 ms depolarizations at different voltages. **(B1)** Measurement of exocytosis (ΔCm) and **(B2)** corresponding Ca^2+^ currents (ICa^2+^) for control (Unexposed, black traces) and 1 d.a.e.(red traces) IHCs obtained during the application of 50 ms pulses at different voltages. Statistical comparisons were made using Two-way ANOVA followed by Holm-Sidak multiple comparisons test. *p < 0.05. **(C)** Analysis of Ca^2+^ dependence of vesicle release in control and exposed IHCs. Gray and red filled circles correspond to the mean exocytic responses triggered by pulses in the range of −50 to −20 mV plotted vs. the mean corresponding Ca^2+^ current integrals for each potential in a double-logarithmic scale for unexposed and 1 d.a.e mice, respectively. Linear regressions were fitted using all the observations (empty gray and red circles) of each group and slopes of the linear fit (*m*) were obtained. Slope comparisons were made using ANCOVA test. **p < 0.01. Data is shown as mean ± standard error of the mean (SEM).

We tested the functional status of IHC neurotransmitter release capacity by evaluating Ca^2+^-triggered fusion of IHC synaptic vesicles from 1 d.a.e. mice and age-matched controls (Figure 4). The noise exposure protocol was designed to target low frequency areas of the cochlea, thus all IHCs recorded in this study were located in the apical coil. Representative traces of Ca^2+^ currents and changes in membrane capacitance (ΔCm) in response to pulses of 50 ms to −50, −30, and +10 mV are shown in Figure 4A. For unexposed mice, the complete curve for potentials between −50 and +30 mV shows the expected bell shape with a maximum ΔCm of 14.24 ± 1.80 fF at −20 mV (Figure 4B1). As expected, IHC Ca^2+^ currents showed a similar relation with Vm (Figure 4B2) with a maximum of −102.97 ± 6.51 pA at −20 mV, in accordance with previous reports (Moser and Beutner, 2000; Johnson et al., 2005). Interestingly, ΔCm in exposed IHCs (*n* = 10) was significantly higher than in unexposed cells (*n* = 9) and the ΔCm vs. V_*m*_ curved peaked at −30 mV with an average of 20.23 ± 3.24 fF representing 58.66% of enhancement (Figure 4B1; *p* = 0.0389, at −30 mV). A significant increase in Ca^2+^ current amplitudes were observed only at +20 and +30 mV steps of noise exposed IHCs (Figure 4B2; *p* = 0.0326, for both potentials) (Kros and Crawford, 1990). Therefore, these results reveal an enhanced neurotransmitter release after noise exposure with unchanged Ca^2+^ currents for voltage pulses <−20 mV, suggesting a noise-induced increase in vesicle release efficiency at the presynaptic ribbon, specifically for stimuli within the physiological range of membrane potentials (Russell and Sellick, 1983; Dallos, 1985). We further investigated the Ca^2+^ dependence of exocytosis for both groups by looking at the relation between ΔCm and Ca^2+^ currents integral (QCa^2+^) (Figure 4C, note the double logarithmic plot), for depolarizing pulses between −50 and −20 mV. For unexposed IHCs, the slope of the linear regression fitted to data was 0.98 ± 0.17, a value that is in agreement with previous reports showing a linear relationship between exocytosis and Ca^2+^ entry for apical IHCs after hearing onset (i.e., an exponent of 1 in the power relation, ΔCm = a I_*Ca*_^*b*^) (Brandt et al., 2005; Johnson et al., 2005). In contrast, noise exposed IHCs showed a significantly higher slope value of 1.74 ± 0.22 (*p* = 0.0079), indicating an increased apparent Ca^2+^ cooperativity for vesicle release.

Having electrophysiological and immunofluorescence data corresponding of the same cochlear regions from the same mice allowed us to estimate how much exocytosis occurs per synaptic ribbon in control and noise exposed IHCs, assuming that CtBP2-Ribeye positive puncta reveal the ribbon sites whereby vesicles would fuse. With steps to −30 mV, in control conditions the ratio of ΔCm/# ribbons is 0.75 fF/ribbon, whereas in noise exposed cells this value increased by 81% to 1.36 fF/ribbon [representing 19 and 34 vesicles/ribbon respectively, considering an intrinsic capacitance of 40 aF for a single vesicle (Grabner and Moser, 2018)]. Therefore, the per synapse increase in vesicle release after noise exposure is even larger than what is shown in Figure 4, which in turn suggests a more Ca^2+^-efficient exocytosis in IHCs after noise exposure.

**FIGURE 5:**
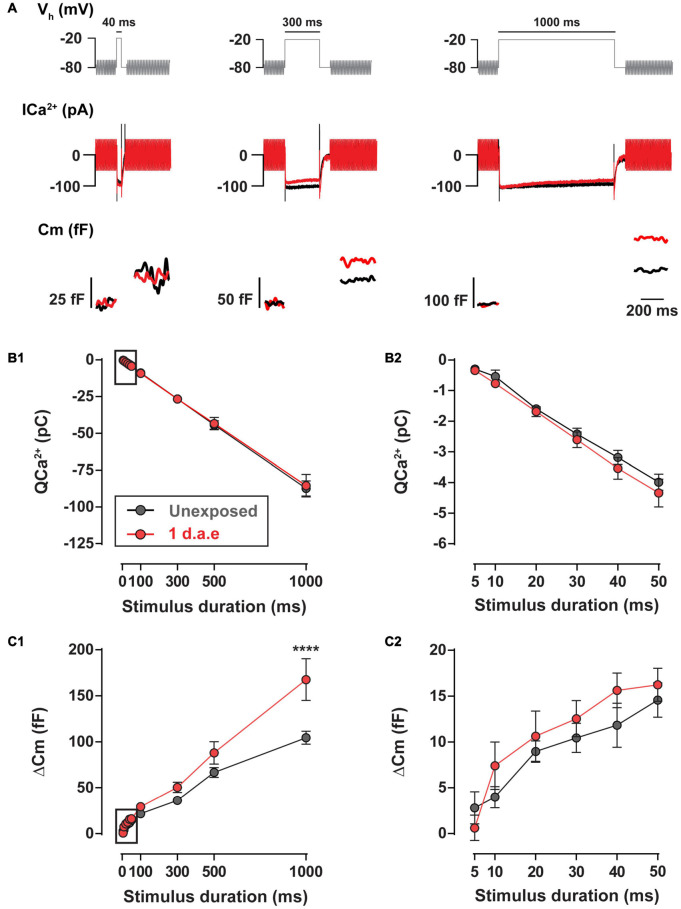
Exocytosis triggered by long pulses is increased 1 d.a.e. to 120 dB noise. **(A)** Voltage protocol (top), Ca^2+^ currents (ICa^2+^) (middle), and membrane capacitance (Cm) (bottom) traces of representative control (Unexposed, black traces) and 1 d.a.e. (red traces) inner hair cells (IHCs) elicited by −20 mV test pulses of 40 ms (left panel), 300 ms (middle panel), and 1,000 ms (right panel). **(B1,C1)** Ca^2+^ current integrals (QCa^2+^) and exocytosis (ΔCm) corresponding to −20 mV pulses of different durations. **(B2,C2)** Expansion of B1 and C1 depicting the calcium entry and exocytosis triggered by −20 mV pulses between 5 and 50 ms. Data is presented as mean ± standard error of the mean (SEM). Statistical comparisons were made using Two-way ANOVA followed by Holm-Sidak multiple comparisons test. ****p < 0.0001.

The high temporal precision of sound encoding relies on the continuous release of synaptic vesicles by IHCs, engaging multiple synaptic vesicle pools (Moser and Beutner, 2000; Johnson et al., 2005; Ruel et al., 2008). We addressed the impact of noise exposure in the kinetics of neurotransmitter release using depolarizing pulses to −20 mV of different durations (Figure 5). No differences in Ca^2+^ entry was found between control and exposed IHCs for the whole range of stimulus durations (10–1,000 ms) (Figure 5B; *p* > 0.05, for all stimulus durations). Using pulse durations of 50 ms, which triggers vesicle release exclusively from the readily releasable pool (RRP) (Moser and Beutner, 2000; Johnson et al., 2005; Ruel et al., 2008), we observed no changes in IHCs exocytosis after noise exposure (Figure 5C2, *p* > 0.05). For 100, 300, and 500 ms pulses we did not find differences in exocytosis (*p* > 0.05), although it was significantly enhanced for 1,000 ms depolarizing pulses (Figure 5C1; *p* < 0.0001), suggesting a noise-induced alteration of the slow secretory component.

Altogether, results in Figures 4, 5 indicate that exposure to loud noise led to a potentiation in the evoked vesicle release from fewer IHC synaptic ribbons with superlinearization in the Ca^2+^ influx-to-release coupling and unaltered Ca^2+^ influx.

### Exocytosis Potentiation Is Absent in Noise Exposed Inner Hair Cell From *Vglut3* Knock-Out Mice

Previous evidence indicated that the activation of AMPA receptors during noising protocols results in SGN terminal swelling (Puel et al., 1991, 1998; Ruel et al., 2000), and also that the absence of glutamate concentration in the lumen of IHCs synaptic vesicles prevents synapse losses (Kim et al., 2019). These observations led us to investigate the consequences of the absence of glutamate signaling on the changes of IHC release mechanisms after noise exposure. To this end, we made use of *Vglut3* deficient mice that lack glutamate release from IHCs resulting in complete deafness, slowly progressing loss of IHC–SGN synapses but with relatively preserved synaptic contacts with SGN in 2 weeks old animals (Ruel et al., 2008; Seal et al., 2008).

**FIGURE 6:**
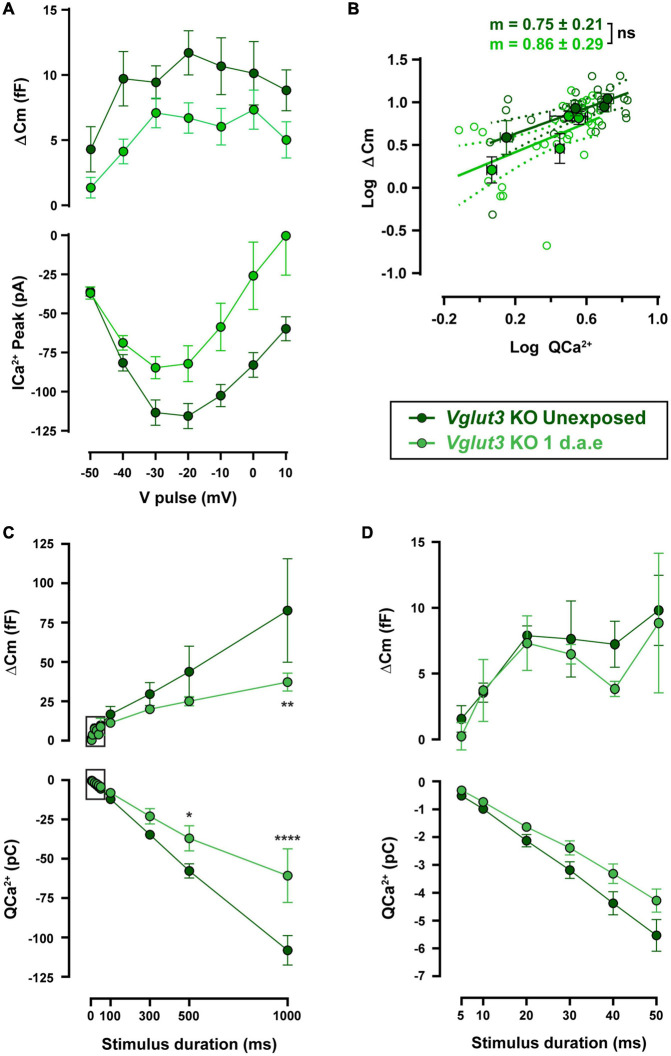
Noise-induced increase in exocytosis capacity is absent in *Vglut3*^*KO*^ mice. **(A)** Measurement of exocytosis (ΔCm, upper panel) and corresponding Ca^2+^ current integrals (QCa^2+^, lower panel) for *Vglut3* knock-out (KO) control (Unexposed, dark green traces) and 1 d.a.e. *Vglut3*^KO^ (green traces) inner hair cells (IHCs) obtained during the application of 50 ms pulses of different voltages. Statistical comparisons were made using Two-way ANOVA followed by Holm-Sidak multiple comparisons test. **(B)** Mean exocytic responses triggered by pulses in the range of −50 to −20 mV plotted vs. the mean corresponding Ca^2+^ current integrals for each potential in a double-logarithmic scale. Linear regressions were fitted as in Figure 2C and slopes of the linear fit (*m*) were calculated. Slope comparisons were made using ANCOVA test. **(C)** Exocytosis (ΔCm) and Ca^2+^ current integrals (QCa^2+^) corresponding to −20 mV pulses of different durations for experimental groups mentioned above (*n* = 6 for both groups). **(D)** Expansion of panel C depicting the exocytosis and calcium entry triggered by −20 mV pulses between 5 and 50 ms. Data is presented as mean ± standard error of the mean (SEM). Statistical comparisons were made using Two-way ANOVA followed by Holm-Sidak multiple comparisons test. *p < 0.05, **p < 0.01, ****p < 0.0001. Data is shown as mean ± SEM.

Ca^2+^-triggered exocytosis was not impaired in *Vglut3*^KO^ IHCs (Ruel et al., 2008). As reported previously, *Vglut3*^KO^ IHCs presented unchanged ΔCm but larger Ca^2+^ influx (see unexposed data from WT and KO cells in Figures 5, 6: ΔCm_WT_ vs. ΔCm*_*Vglut*__3_*_KO_: *p* > 0.05 and QCa^2+^_WT_ vs. QCa^2+^_Vglut3 KO_: *p* < 0.05 for all pulse durations). *Vglut3*^KO^ mice were noise exposed with the same protocol as used for WT, and at 1 d.a.e cochleas were dissected and IHCs were patch-clamped. Figure 6A shows ΔCm and Ca^2+^ currents amplitudes elicited by voltage pulses between −50 and +10 mV, for both unexposed (*n* = 8) and 1 d.a.e. IHCs (*n* = 7). In contrast to our observations in WT mice (Figure 4), no increase in exocytosis was observed after noise exposure in *Vglut3*^KO^ IHCs. Instead, ΔCm and Ca^2+^ influx were reduced, but not significantly (Figure 6A, 44% reduction for ΔCm and 41% for Ca^2+^ currents, *p* > 0.05 for all stimulus voltages). No differences were found in the slope of the linear fit to the ΔCm vs. QCa^2 +^ plots with values of 0.75 ± 0.21 and 0.86 ± 0.29 for control and exposed, respectively (Figure 6B, *p* = 0.78), indicating that no changes occurred in the Ca^2+^-release coupling after exposure of *Vglut3*^KO^ IHCs.

Functional kinetic components were also evaluated in noise exposed and control *Vglut3*^KO^ mice, as shown in Figures 6C,D. No differences were found in either ΔCm or QCa^2 +^ for pulses up to 50 ms, indicating that RRP vesicles were not affected by exposure (Figure 6D). Longer pulses in exposed *Vglut3*^KO^ IHCs reflected a reduced capacity to release neurotransmitter (Figure 6C) that was accompanied by a smaller Ca^2+^ influx. Considering that the slope in the ΔCm vs. QCa^2 +^ plot did not change after noise exposure in *Vglut3*^KO^ cells (Figure 6B), and that the reduction in exocytosis is similar to the decrease in Ca^2+^ currents amplitude (∼40% reduction in average for ΔCm and Ca^2+^ currents in Figure 6A and a reduction of 36.5% for ΔCm and 30.5% for QCa^2 +^ in Figure 6C), it can be proposed that noise exposure did not produce changes in release mechanisms of *Vglut3*^KO^ IHCs downstream of Ca^2+^ influx. This observation contrasts to what was observed in WT cells after acoustic trauma, indicating that glutamate release has an important role in the reconfiguration of the release-Ca^2+^ influx coupling in IHCs of exposed mice.

## Discussion

The nature of the injury that acoustic overexposure produces to the inner ear has been intensively investigated over decades (Spoendlin, 1971; Robertson, 1983). Nonetheless, the physiological consequences of noise exposure to IHC synaptic function have been less investigated. In our current study, we found that noise exposure triggers changes in the mechanisms of exocytosis. These changes are likely dependent on glutamate signaling as they were absent in *Vglut3*^KO^ mice.

### Noise Exposure Potentiated Exocytosis and Altered Coupling to Ca^2+^ Influx

As described previously for intense exposures of octave-band noise, damage to the cochlea is typically found in characteristic frequency regions half an octave above the noise band and in more basal regions (Liberman and Kiang, 1978; Robertson and Johnstone, 1980; Kujawa and Liberman, 2009; Kim et al., 2019). In this study, the frequency band of our noise exposure protocol was lowered in order to target the most apical region of the cochlea where IHCs are more accessible to electrophysiological recordings and mechanisms of release have been thoroughly studied (Moser and Beutner, 2000; Brandt et al., 2005; Johnson et al., 2005; Goutman and Glowatzki, 2007). As shown in Figure 3, significant synapse loss was found in exposed IHCs at the 8–12 kHz cochlear region that was used to evaluate exocytosis by capacitance measurements. Although the number of presynaptic active zones (CtBP2 colocalized with Ca_V_1.3) was reduced 1 d.a.e., no reduction in exocytosis was observed in IHCs. Rather, exocytosis increased by as much as ∼60% compared to unexposed IHCs (e.g., −30 mV pulses in Figure 4; 1,000 ms pulses in Figure 5). Considering that cellular capacitance measurements detect changes in exocytosis across all active zones of an IHC, and that the number of active zones was reduced at 1 d.a.e., the enhancement per active zone was ∼81% for steps to −30 mV. Therefore, after noise exposure, IHC exocytosis appears to be more Ca^2+^ efficient, given that no significant changes in Ca^2+^ influx were observed (Figure 4B2).

On the other hand, our results also indicate that after noise exposure the relationship between ΔCm and QCa^2 +^ escapes the typical linearity found in unexposed IHCs from the cochlear apex (Brandt et al., 2005; Johnson et al., 2005; Goutman and Glowatzki, 2007), suggesting that physical coupling between vesicles and Ca^2+^ entry is more “loose.” It is possible that Ca^2+^ channels redistributed such that a constant number of channels per cell would be shared among fewer synapses, resulting in a larger average distance between channels and vesicles. However, our Ca_V_1.3 immunostainings do not show evidence for puncta with larger volumes which would be expected under this scenario assuming a constant packing density. Alternatively, vesicles could be re-positioned around Ca^2+^ channels after noise exposure. Changes in the accumulation of intracellular membranes and counts of synaptic vesicles have been observed recently in afferent synapses of IHCs after noise exposure (Bullen et al., 2019). The RRP size, as determined in Figure 5, does not show changes compared to control using pulses to −20 mV. However, results in Figure 4 using pulses of 50 msec at −30 mV might indicate a higher number of available vesicles per active zone after exposure. Changes in the vesicular Ca^2+^ sensor due to acoustic trauma could also underlie these phenomena if, for example, the expression levels of otoferlin or synaptotagmin-IV are affected (Beurg et al., 2010; Johnson et al., 2010).

It was recently shown that despite the linear relation between Ca^2+^ influx and release, measured across all synapses within a given IHC, individual active zones can be heterogeneous in the mode of excitation-secretion coupling, with some showing a highly supralinear release mechanism (Heil and Neubauer, 2010; Özçete and Moser, 2020). If one hypothesized that synapses with linear coupling are more vulnerable to noise and the first to be functionally altered or lost, it could be proposed that the remaining ones would “push” the ensemble coupling relation to a more supralinear mode. According to Özçete and Moser (2020), “linear synapses” are more likely to occur on the pillar side of the IHC where contacts with high spontaneous rate SGNs are typically found, at least in cats (Liberman, 1982). However, previous evidence (from guinea pigs) indicates that the low spontaneous rate SGNs are the most sensitive to noise and the first to die (Furman et al., 2013). More work is needed to show if the higher vulnerability of “linear synapses” is a true physiological phenomenon.

We observed drastic DPOAEs threshold elevation that persisted to at least 14 d.a.e., indicating a highly impaired OHC function after exposure to noise. These results point to a more permanent injury to OHCs, although very few were lost in the 8–12 kHz region. Thus, the large elevation of DPOAEs thresholds we observed might be caused by alterations in the functional properties of surviving OHCs, such as, mechanotransduction due to stereocilia disarray, or disruption of the physical association between the tectorial membrane and the stereocilia (Saunders et al., 1985). It has been recently hypothesized that hi-level noise exposure protocols producing permanent thresholds elevations from OHC damage could reduce excitation to the IHC and thus reduce synaptopathy relative to more moderate exposures (Fernandez et al., 2020). Thus, it can be argued that at least part of the synaptic changes observed throughout our study result from OHCs dysfunction (see below).

The effects of noise trauma on synaptic function may depend on the animal species or strain of mice, the details of the exposure protocol (SPL, duration, frequency), or the age of the animals at exposure. In a recent study in mature CBA/CaJ mice, it was shown that after moderate noise exposure (2–20 kHz at 98 dB SPL) causing only temporary threshold shifts, sustained exocytosis and vesicle replenishment was reduced (Liu et al., 2019). In the current study we exposed C57BL/6 mice at P15-16. Thus, it is likely that acoustic overexposure at this early stage could produce stronger inner ear effects than those already reported in mature animals. Several studies have shown that exposure to loud noise early during development greatly alters the functional maturation of the auditory system (Sanes and Bao, 2009; Lauer and May, 2011). More work is needed to determine if our findings in young mice can be replicated in more mature animals, or if the same exposure to adult mice would have different effects on exocytosis.

### Potentiation of Inner Hair Cells Exocytosis as a Compensatory Mechanism?

Results from Figures 4, 5 indicate that IHCs present a potentiated exocytotic capacity when evaluated 1 d.a.e. It can be proposed that this enhancement is related to the loss of synapses after noise exposure, suggesting that some form of homeostatic plasticity occurs at IHCs afferent synapses to compensate for the loss of release sites (Delvendahl and Müller, 2019). An alternative interpretation of these results is that the exocytosis enhancement is not due to synaptopathy but rather a compensatory adaptation of IHC synapses due to reduced mechanical drive to IHCs resulting from OHC damage (see Figures 1, 2). It should be emphasized that homeostatic plasticity is not rare in the auditory system. The endbulbs of Held of noise-reared mice (with non-damaging acoustic stimuli) show an increase in the number of release sites, together with a reduction in release probability (Ngodup et al., 2015). Adaptations in neuronal mechanisms have also been described as a consequence of auditory deprivation (Oleskevich and Walmsley, 2002; Kuba et al., 2010; Zhuang et al., 2017). Thus, it could be hypothesized that the auditory system responds with homeostatic adaptations to drastic changes in the level of acoustic input, and that these changes would operate to maintain a well-balanced synaptic signaling. Under this scenario, IHCs release more neurotransmitter to compensate for the synapse loss and/or the reduced excitation due to OHC dysfunction.

It seems reasonable to think that a mechanism to adapt to environmental changes in sound intensity provided evolutionary fit to vertebrate’s auditory systems. However, it could be contradictory that IHCs underwent a process of release potentiation after noise exposure if excessive glutamate is potentially harmful for SGNs (Puel et al., 1991). It is important to consider that overintense stimuli typically used to noise-expose mice is an experimental manipulation that would hardly be found in the natural history of these animals, only after the appearance of big cities and industrialization. Thus, evolutionary thinking may not apply to interpret biological responses to this type of treatment.

### No Change in Exocytosis From Noise-Exposed Inner Hair Cells of *Vglut3*^KO^

It has been suggested that sound overexposure leads to excessive release of glutamate because dendrite damage is very similar to that seen after perfusion of the cochlea to glutamate receptor agonists (Puel et al., 1991, 1995). Noise exposure may also activate several damage pathways by releasing adenosine, aspartate and proinflammatory cytokines (Kurabi et al., 2017). Studies with the *Vglut3*^KO^ mice have shown that synaptic release of glutamate is required for noise-induced cochlear synaptopathy (Kim et al., 2019). We found that noise exposure produced a reduction of vesicle exocytosis in mice with genetic ablation of *Vglut3*, suggesting that noise-induced increase in IHC exocytosis in WT may be dependent on synaptic release of glutamate from IHCs or some other function of *Vglut3* transporter. The mechanism of glutamate-dependent potentiation of vesicle release is not clear but might involve local circuits such as retrograde glutamate signaling through mGluRs, which have been recently detected functionally and anatomically at IHC ribbon synapses (Ye et al., 2017; Klotz et al., 2019). Alternatively, glutamate dependent activation of the auditory brain by the auditory nerve may have resulted in efferent activity that caused the potentiation of exocytosis from IHCs. This possibility could be tested in future studies using mice that lack efferent signaling. Taking the results in WTs and KOs together, it can be speculated that the reduction in vesicle release and Ca^2+^ currents in exposed *Vglut3*^KO^ could be triggered by factors other than glutamate. One possible mechanism is that large Ca^2+^ influx during exposure triggers a spike in the concentration of reactive oxygen species and/or activate intracellular signaling cascades that mediate cell stress resulting in a reduced exocytosis capacity (Kurabi et al., 2017). In WT IHCs these effects might be out compensated by the glutamate mediated enhancement.

In summary, our results show that noise exposure triggers an increase in IHC exocytosis together with a change in the coupling between Ca^2+^ influx and exocytosis. If this is a compensatory response to reduced cochlear amplification, or alternatively due to the synapse loss, is still an open question. Experiments in *Vglut3*^KO^ mice indicate that this phenomenon is dependent on glutamate signaling.

## Data Availability Statement

The raw data supporting the conclusions of this article will be made available by the authors, without undue reservation.

## Ethics Statement

The animal study was reviewed and approved by the Animals Studies Committee of Washington University in St. Louis, INGEBI, and Facultad de Medicina UBA.

## Author Contributions

LB, MR, MG-C, and JG contributed with the conception and design of the study. LB and SP acquired the data. LB, MR, MG-C, SP, and JG analyzed the data. All authors contributed with manuscript writing and revision.

## Conflict of Interest

The authors declare that the research was conducted in the absence of any commercial or financial relationships that could be construed as a potential conflict of interest.

## Publisher’s Note

All claims expressed in this article are solely those of the authors and do not necessarily represent those of their affiliated organizations, or those of the publisher, the editors and the reviewers. Any product that may be evaluated in this article, or claim that may be made by its manufacturer, is not guaranteed or endorsed by the publisher.
