# Supplementary material for: Noise Exposure Potentiates Exocytosis From Cochlear Inner Hair Cells
Source: Front Synaptic Neurosci. 2021 Sep 29;13:740368. doi: 10.3389/fnsyn.2021.740368 (PMC8511412; doi:10.3389/fnsyn.2021.740368)
Supplement: Supplementary file 1 [file Table_1.DOCX]

Supplementary Material

| **Dunn's multiple comparisons test** | **Adjusted P-Value** | **Summary** |
| --- | --- | --- |
| 5.6 kHz Unexposed vs. 5.6 kHz NE+1d | 0.0010 | ** |
| 5.6 kHz Unexposed vs. 5.6 kHz NE+14d | 0.0237 | * |
| 8 kHz Unexposed vs. 5.6 kHz NE+1d | 0.0058 | ** |
| 8 kHz Unexposed vs. 5.6 kHz NE+14d | 0.0036 | ** |
| 11.2 kHz Unexposed vs. 5.6 kHz NE+1d | 0.0030 | ** |
| 11.2 kHz Unexposed vs. 5.6 kHz NE+14d | 0.0049 | ** |
| 16 kHz Unexposed vs. 5.6 kHz NE+1d | 0.0167 | * |
| 16 kHz Unexposed vs. 5.6 kHz NE+14d | 0.0012 | ** |
| 22.6 kHz Unexposed vs. 5.6 kHz NE+1d | 0.0038 | ** |
| 22.65 kHz Unexposed vs. 5.6 kHz NE+14d | 0.0055 | ** |
| 32 kHz Unexposed vs. 5.6 kHz NE+1d | 0.0018 | ** |
| 32 kHz Unexposed vs. 5.6 kHz NE+14d | 0.0021 | ** |
| 45.2 kHz Unexposed vs. 5.6 kHz NE+1d | 0.0001 | *** |
| 45.25 kHz Unexposed vs. 5.6 kHz NE+14d | 0.0001 | *** |

**Supplementary Table 1.** List of the p-values obtained after Dunn’s Multiple Comparisons Test of the ABR Threshold data in Figure 1B. The first column indicates the groups that were compared whereas the second column shows the exact p-value of that statistical comparison. Last column refers to the code of asterisks used in the graph to denote the level of significance of a p-value.
